# Insights Into Mutations Induced Conformational Changes and Rearrangement of Fe^2+^ Ion in *pncA* Gene of *Mycobacterium tuberculosis* to Decipher the Mechanism of Resistance to Pyrazinamide

**DOI:** 10.3389/fmolb.2021.633365

**Published:** 2021-05-20

**Authors:** Asma Sindhoo Nangraj, Abbas Khan, Shaheena Umbreen, Sana Sahar, Maryam Arshad, Saba Younas, Sajjad Ahmad, Shahid Ali, Syed Shujait Ali, Liaqat Ali, Dong-Qing Wei

**Affiliations:** ^1^Department of Bioinformatics and Biological Statistics, School of Life Sciences and Biotechnology, Shanghai Jiao Tong University, Shanghai, China; ^2^Department of Botany, University of Okara, Okara, Pakistan; ^3^The Islamia University of Bahawalpur, Bahawalpur, Pakistan; ^4^Government College University Faisalabad, Sahiwal, Pakistan; ^5^University of Education, Lahore, Pakistan; ^6^Department of Health and Biological Sciences, Abasyn University, Peshawar, Pakistan; ^7^Center for Biotechnology and Microbiology, University of Swat, Swat, Pakistan; ^8^Department of Biological Sciences, National University of Medical Sciences, Islamabad, Pakistan; ^9^Peng Cheng Laboratory, Shenzhen, China; ^10^State Key Laboratory of Microbial Metabolism, Shanghai-Islamabad-Belgrade Joint Innovation Center on Antibacterial Resistances, Joint International Research Laboratory of Metabolic and Developmental Sciences, School of Life Sciences and Biotechnology, Shanghai Jiao Tong University, Shanghai, China

**Keywords:** PZA, simulation, mutations, PCA, free energy

## Abstract

Pyrazinamide (PZA) is the first-line drug commonly used in treating *Mycobacterium tuberculosis (Mtb)* infections and reduces treatment time by 33%. This prodrug is activated and converted to an active form, Pyrazinoic acid (POA), by Pyrazinamidase (PZase) enzyme. *Mtb* resistance to PZA is the outcome of mutations frequently reported in *pncA*, *rpsA*, and *panD* genes. Among the mentioned genes, *pncA* mutations contribute to 72–99% of the total resistance to PZA. Thus, considering the vital importance of this gene in PZA resistance, its frequent mutations (D49N, Y64S, W68G, and F94A) were investigated through in-depth computational techniques to put conclusions that might be useful for new scaffolds design or structure optimization to improve the efficacy of the available drugs. Mutants and wild type PZase were used in extensive and long-run molecular dynamics simulations in triplicate to disclose the resistance mechanism induced by the above-mentioned point mutations. Our analysis suggests that these mutations alter the internal dynamics of PZase and hinder the correct orientation of PZA to the enzyme. Consequently, the PZA has a low binding energy score with the mutants compared with the wild type PZase. These mutations were also reported to affect the binding of Fe^2+^ ion and its coordinated residues. Conformational dynamics also revealed that β-strand two is flipped, which is significant in Fe^2+^ binding. MM-GBSA analysis confirmed that these mutations significantly decreased the binding of PZA. In conclusion, these mutations cause conformation alterations and deformities that lead to PZA resistance.

## Introduction

Pyrazinamide (PZA), along with isoniazid (INH) and rifampin (RIF) is a very effective and fast therapy against persistent bacilli (Mitchison, 1985; Aggarwal et al., 2018). Pyrazinamide (PZase) encoded by the pncA gene of *Mycobacterium tuberculosis* (Mtb) transform this prodrug to pyrazinoic acid (POA). POA inhibits the proliferation of latent Mtb at very low pH values (Zhang and Mitchison, 2003; Malik et al., 2019). Studies have shown that resistance is developed against PZA due to mutations in three genes: *pncA, panD*, and *rpsA*, among which *pncA* gene mutations contribute to 72–99% resistance against PZA (Mitchison, 1985; Akhmetova et al., 2015; Miotto et al., 2017).

Mutations in the *pncA* gene have been mapped both in the coding as well as promoter region (Lemaitre et al., 2001; Miotto et al., 2014; Maningi et al., 2015). Recent investigations indicated that Pzase activity is affected due to mutations in D49A, Y64S, W68G and F94A positions (Miotto et al., 2014). The mentioned mutations have been shown to affect enzyme functionality drastically, and together with other reported mutations, influence protein structure integrity, solubility, function stability, and rate of expression (Petrella et al., 2011). More recently, novel pncA mutations are being described as liable to cause PZA resistance (Tan et al., 2014; Junaid et al., 2018).

The crystallographic structure of apo pyrazinamidase has been reported comprising six β-sheets covered by α-helices. This enzyme has metal and substrate binding sites. Iron (Fe^2+^ ion), histidine (His51, His57, and His71), and aspartate (Asp49) residues are part of the metal-binding site, whereas Asp8, Lys96, and Cys138 make the catalytic triads (Chaturvedi and Shrivastava, 2005; Petrella et al., 2011).

Computational approaches are now in routine to decipher mutations mediated biological mechanisms responsible for neutralizing the action of potent drugs. This atomic-level understanding holds great potential in de nova drug design and as such, speeds up novel drug discovery. In particular, advancements in molecular dynamics simulations allows scientist to analyze protein dynamics in environmental milieu replica of real biological cells (Khan et al., 2018a,b, 2019a, 2020a). It has been noticed that binding of PZA to the PZase enzyme altered protein’s conformation, which is valuable data-keeping their importance in the quest of novel drug design. Likewise, MD simulations made it possible to study conformational variations in the three-dimensional structure of proteins that may arise following mutation(s) in the sequence (Khan et al., 2019c, 2020b,f). Thus MD simulations decrease time, costs and resources by reducing the number of cases for which experimental evaluation is required (Dolatkhah et al., 2017). We also investigated the molecular mechanism behind the resistance caused by D49N, Y64S, W68G, and F94A mutations (Stoffels et al., 2012; Miotto et al., 2014; Wan et al., 2020). Furthermore, extensive post-simulation analyses were employed to get insights into the atomic level with an ultimate objective to design novel chemical structures that can be effectively used in drug-resistant TB infections—with minimum side effects.

## Materials and Methods

### PZase and PZA Structure Retrieval

The 3D structure of Mtb PZase (accession ID: 3PLI) and PZA (accession ID CID1046) were retrieved from the PDB databank (Rose et al., 2016) and PubChem (Kim et al., 2019) respectively. Water molecules were removed from the protein structure before starting downward analyses. As specific mutant structures of the enzyme were not available, mutations were introduced in the enzyme structure using PYMOL (DeLano, 2002) at particular locations.

### Molecular Docking

Energy minimization steps were performed for PZA structure in Open Babel using Universal Force Field (Dallakyan and Olson, 2015). The ligand was optimized with default steepest descent and conjugate gradient algorithms in UCSF Chimera (Goddard et al., 2005). Docking was done in the PatchDock server, where binding conformation clusters were set at RSMD of 4.0 Å (Schneidman-Duhovny et al., 2005). Conformations with the lowest binding score were processed for molecular dynamics simulation using AMBER18 software (Wang et al., 2001; Case et al., 2005).

### Impact of Mutations on Protein-Drug Interaction and Stability

Mutations’ effect on protein thermodynamic stability was evaluated using mCSM^1^ (Pires et al., 2013). The server utilizes graph-based signatures to predicts structural stability impact caused by mutation. mCSM accepts PDB files as input and a list of mutations to predict their effect on protein stability.

### Molecular Dynamics Simulation

AMBER18 package was used to perform extensive MD simulations. This was done to investigate the stability of the PZA at the active site of both normal and mutant PZase. Parameters of protein were generated through ff14SB force field, and ligand preparation was done via Amber general force field (GAFF) (Wang et al., 2001; Case et al., 2005). MD simulations were performed for all five systems, including one wild (WT) and four mutants (D49N, Y64S, W68G, and F94A). Each system is solvated in TIP3P water box. Counter ions were added to each system to get charge neutralization. Afterward, two-step energy minimization procedure was adopted; (i) steepest decent minimizations of 6,000 cycles and (ii) conjugate gradient minimization of 3,000 cycles was applied on each system to remove steric clashes and allow system relaxation. Complexes were then heated to 300 K for 0.2 ns, followed by systems equilibration for 2 ns at 300 K. Temperature hold was achieved *via* Langevin thermostat (Zwanzig, 1973). For all systems, MD simulations production run was completed on GPU supported PMEMD code for 100 ns, and each simulation was repeated three times. Long-range electrostatic interactions (Darden et al., 1993; Essmann et al., 1995; Toukmaji et al., 2000) were detected with the particle mesh Ewald method using a cutoff distance of 10.0 Å. SHAKE method was applied for covalent bond treatment (Kräutler et al., 2001) (Salomon-Ferrer et al., 2013). CPPTRAJ and PTRAJ (Roe and Cheatham, 2013) packages in AMBER18 were considered for trajectories analysis.

### Principal Component Analysis

Principal component analysis was utilized to measure structural fluctuations within the protein of all used complexes (Amadei et al., 1993). CPPTRAJ package calculated the covariance matrix based on Cα coordinates. Eigenvectors and eigenvalues estimation was performed by diagonalizing the covariance matrix, and these values indicate motion direction and fluctuation, respectively. In total, 5000 frames from each system MD trajectories were used to get PCA calculations. The plotting performed on PC1 and PC2 was used for motion monitoring. The lowest energy stable state was determined by the free energy landscape (FEL) and is indicated by deep valleys on the plot, whereas the intermediate state is shown by boundaries between deep valleys (Hoang et al., 2004). In this study, FEL calculations based on PCI and PC2 were obtained by the following equation:

ΔG(PC1,PC2)=-KBTlnP(PC1,PC2)

Where KB indicates Boltzmann constant, PC1 and PC2 were used to estimate the reaction coordinates, and probability distribution P of the system is shown along PC1 and PC2.

### Binding Affinity Estimation

PZA binding free energy with PZase (native and mutants) was estimated through MMPBSA.py script of AMBER over 500 snapshots of simulation trajectories (Miller et al., 2012; Mishra and Koča, 2018). The equation given below is used for binding free energy calculations

$$\Delta G_{\text{bind}}=\Delta G_{\text{complex}}-\left[\Delta G_{\text{receptor}}+\Delta G_{\text{ligand}}\right]$$

where ΔG_*bind*_, ΔGcomplex, ΔGreceptor, and ΔGligand indicate net binding free energy, binding free energy of the complex, protein, and ligand, respectively. The following equation was used to calculate the value of each component:

$$G=G_{\text{bond}}+G_{\text{ele}}+G_{\text{vdW}}+G_{\text{pol}}+G_{\text{npol}}$$

where the energy of bonds, electrostatic, van der Waals interactions, the polar and non-polar contributions are shown by the Gbond, Gele, GvdW, Gpol, and Gnpol, respectively. Whereas Gpol and Gnpol were calculated by the generalized Born (GB) implicit solvent method with SASA.

## Results

### Mutant PZase Structural Modeling and Docking With PZA

**FIGURE 1:**
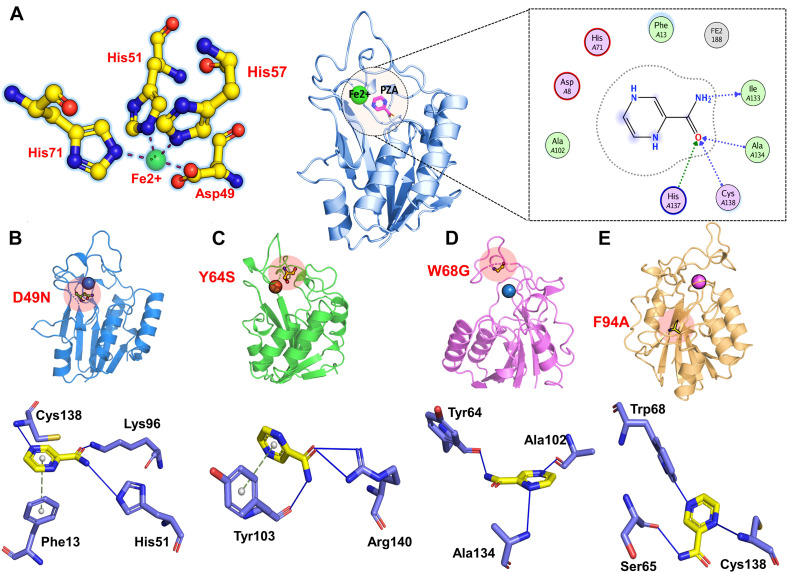
3D structure of the PZase along with the PZA drug and the Fe^2+^ metal shown in the circle. The figure also shows the binding of Fe^2+^ ion and PZA drug to the wild type **(A)**, D49N **(B)**, Y64S **(C)**, W68G **(D)**, and F94S **(E)**.

The PZase apo structure (available as crystal structure) with ID: 3PL1 was retrieved from the protein databank and subjected to mutagenesis module in the PyMOL software where D49N, Y64S, W68G, and F94A mutants were created. Before molecular docking, all the structures were minimized by removing bad contacts from newly mutated residues as well as other residues. Following the minimization process, the docking process was completed blindly. Docking results suggested that our docking protocol is reliable, as indicated by the involvement of similar residues in interaction, as reported by a previous study (Junaid et al., 2018, 2020). Two residues such as His137 and Cys138, were reported to be involved in hydrogen bonding interactions with the oxygen of PZA. In the present study, similar results were obtained. The docking score of all complexes, including wild type and mutants, are tabulated in Table 1. The more negative binding energy implies better PZase-PZA intermolecular complementarity and higher binding affinity in contrast to the positive binding energy. The complex structure of wild type PZase and the PZA and its interaction pattern are given in Figure 1A. The docking score of PZA with both wild type and mutants PZase is in the following order: wild type (−5.21 kcal/mol), D49N (−4.75 kcal/mol), Y64S (−4.1 kcal/mol), W68G (−4.51 kcal/mol) and F94A (−4.18 kcal/mol). This data suggests that the PZA drug has a higher binding affinity for the wild type PZase enzyme in contrast to the mutants. Among the mutants, the lowest binding affinity of the PZA drug was noticed for Y64S and F94A. There is a high possibility that the mutations alter the active pocket conformation and thus not allowing proper PZA binding. The binding interaction pattern of each complex is given in Figures 1–E. MD simulations were performed on top scorer conformations to ascertain the effect of the mutation on the PZase structure as well as its binding with PZA.

**TABLE 1:** Molecular docking scores of the wild and mutant complexes. The mCSM predicted stability changes upon mutation. All the energies are given in kcal/mol.

S. No	Complex	Docking score	Predicted ΔΔG	Outcome
1.	Wild	−5.21	00	–
2.	D49N	−4.75	−2.00	Highly destabilizing
3.	Y64S	−4.1	−2.2	Highly destabilizing
4.	W68G	−4.51	−3.14	Highly destabilizing
5.	F94A	−4.18	−2.94	Highly destabilizing

### Dynamics Characterization of Wild Type and Mutant Complexes

Mutations were found to confer instability in enzyme structure as predicted by mCSM web server and also by RMSD plots from a triplicate run of 100 ns MD simulations. mCSM server predicts the impact of each substitution by forecasting the change in conformational energy. As given in Table 1, it can be easily pointed that the given mutations induced greater instability compared to the wild type and hence classified as highly destabilizing. Among the four mutants, it was observed that W68G has a profound destabilizing effect on the PZase enzyme with ΔG of −3.14 kcal/mol. This was followed by F94A mutation that contributes to enzyme destabilizing change of −2.94 kcal/mol (Miotto et al., 2017). Among others, the predicted destabilizing energy change for Y64S is −2.2 kcal/mol whereas, for D49N, the energy change is 2.0 kcal/mol. These findings are in line with the docking score of the systems and together, both analysis demonstrated the mutations are responsible for the change of PZase active pocket conformation, thus destabilize the binding network of the PZA drug, as can be seen in Figure 1.

**FIGURE 2:**
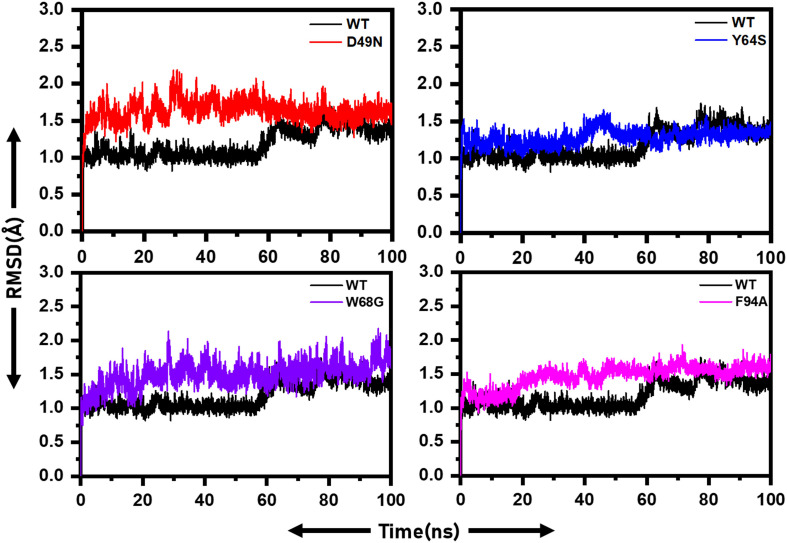
RMSD of wild and mutants’ complexes. RMSD of each mutant is superimposed on to the RMSD of the wild type. The X-axis shows the simulation time in nanoseconds, while the y-axis shows the RMSD in Angstrom.

For the stability assessment of each system, Cα atoms root-mean-square deviation (RMSD) was calculated based on simulated trajectory. The WT system reached an equilibrium state up to 60 ns, followed by a minor RMSD increased up to a maximum of 1.5 Å. Later, the RMSD continued over 1.5 Å with insignificant fluctuation (Figure 2). The D49N mutant system reached an equilibrium state of 2 Å in the first 20 ns, and then the RMSD fluctuated high throughout the simulation time due to system instability compared to WT. The Y64S system, like the WT gained equilibrium in the 50 ns and remained stable with slight fluctuations in the RMSD. The W68G system is in stable conformation till 30 ns with RMSD of 2.2 Å, then retained with RMSD at 1.5 Å and fluctuating slightly from the WT for the rest of time. The F94A system gains equilibrium in the first 10 ns and afterward showing minor fluctuations up to 2 Å. This unstable dynamics behavior of the mutants supports the enzyme conformation changes upon mutations to show resistance against PZA. Further inspection of Cα-RMSD rise for mutants compared to the WT showed that the D49N, Y64S, W68G, and F94A might weaken the active site residues interactions with the PZA. The RMSD of the mutant complexes is comparable with the wild type in terms of RMSD value, but the destability justifies that the different convergences at different intervals faced by the mutant structures but not in the wild type. This explanation of the wild and mutant complexes elucidates that due to small protein, the systems have reached the equilibrium point earlier. Furthermore, it can also be seen that the wild type reached the stability at 1.0Å; however, the other systems gained the equilibrium at ∼1.5Å, which shows the mutations induced structural perturbation in mutant complexes. The RMSD results for the other two replicates are given in Supplementary Figures 1, 2.

**FIGURE 3:**
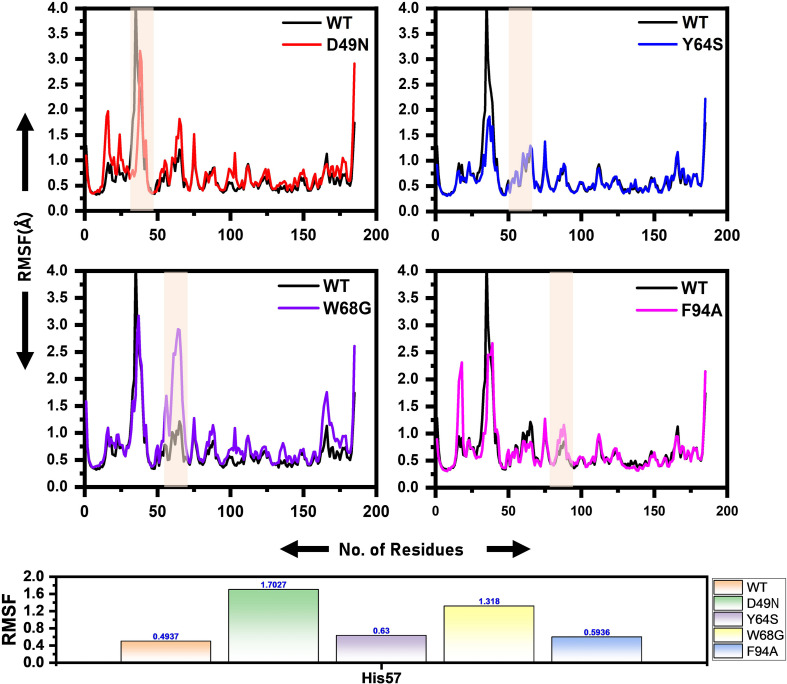
RMSF of the wild and mutants’ complexes. The RMSD of each mutant is superimposed over RMSF of the wild type. The x-axis shows residues number, while the y-axis shows RMSF in Angstrom. Shadowed regions depict enzyme amino acids stretch highly affected by the mutation.

Local fluctuations due to mutations were examined through Cα, root-mean-square fluctuation (RMSF). Residues fluctuation was noted significantly in the mutant systems compared to the WT. WT system fluctuates at the N-terminus. The D49N mutant system reveals several point fluctuations as compared to WT and other mutations. The RMSF high fluctuation from the WT discloses that the mutations greatly affect the binding of the drug to the active site of the protein. The flexibility of the mutants may justify the binding differences, which can be better revealed by exploring the binding affinity differences. In the case of the mutants, the specific fluctuations at the site of the mutation can be easily distinguished. The RMSF of all the systems is given in Figure 3. The RMSF results for the other two replicates are shown in Supplementary Figures 3, 4.

**FIGURE 4:**
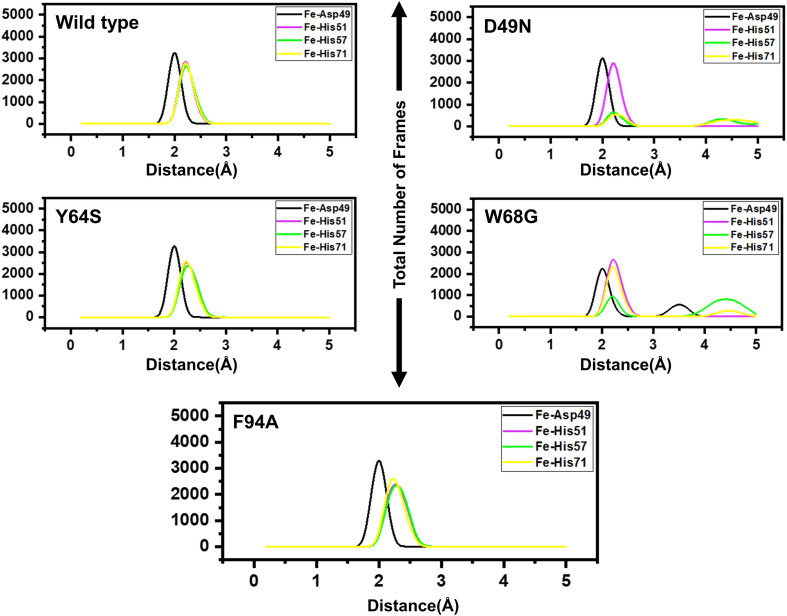
Distances between the Fe^2+^ ion and its coordinating four residues. Furthermore, within each figure inside, there is a legend that shows the distance between Asp49 [O], His51[N], His57[N], His71[N] and Fe^2+^ ion. Each residue from the metal coordinates is differently colored.

Figure 4 presents broader distance distributions in mutants in contrast to WT, indicating more conformation dynamics in the former systems. As three residues: His51, His57, and His71 form a catalytic triad, it is important to understand the effect of these substitutions on the triad dynamics. It can be seen that the wild type, Y64S, and F94A showed a similar pattern of dynamics, while the D49N and W68G possess different triad distance network dynamics. Distinct changes of His57 is due to the loop harboring this residue. Fe^2+^ ion disturbance may reduce PZase activity and may explain the resistance phenomenon of these mutations. This effect was also confirmed by calculating the distance between PZA and the PZase. Supplementary Figure 5 shows that the distance between the wild type and the PZA is conserved, and the average distance reported was 8Å. However, this distance significantly fluctuates in the case of D49N, W68G, and F94A. While in the case of Y64S, the distance between the PZA and the receptor molecule remained somewhat similar to the wild type. Thus, these results also confirm that mutations have induced structural destabilization and favor PZA unbinding due to their weak attachment.

Furthermore, we also calculated *Rg* to estimate the compactness of each system. The calculated Rg for each complex is given in Supplementary Figures 6–8. The results show that D49N is less compact than the wild type. For D49N initially, the higher Rg was observed, which then decreased; however, similar pattern of increasing and decreasing was experienced until 100 ns. In the case of Y64S, the Rg pattern was comparable with the wild type, but at 40–60 ns the Rg converged and a similar pattern was also observed between 95–100 ns.

Similarly, W68G systems were significantly affected. The Rg value significantly increased, and the average Rg was reported to be 15.6Å. The results of F94A and Y64S are comparable. No significant convergence was observed; however, at different intervals, the Rg increased.

### Dimensionality Reduction and Clustering the Protein Motions

**FIGURE 5:**
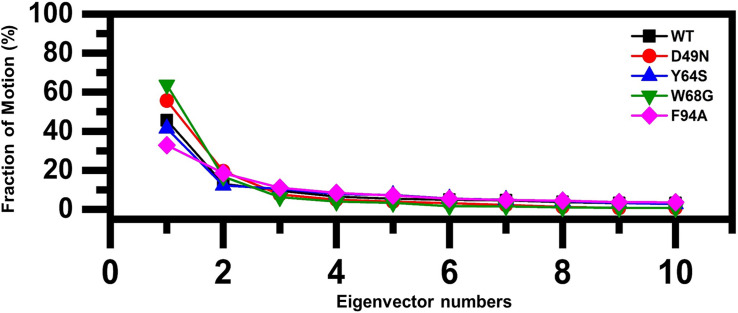
Fractions of the first ten eigenvectors. Using the MD trajectory, the fraction of motions is calculated and given in percentage against the eigenvector numbers.

To understand the protein motion and cluster the related structural frames, PCA was performed. PCA is a mathematical method that transforms several correlated variables into smaller uncorrelated variables called principal components. To comprehensively understand the impact of the substitution on the protein motion initially, the eigenvectors were calculated and presented in Figure 5.

As given in Figure 5, the first three eigenvectors showed significant variations while rest of the eigenvectors showed localized fluctuations. It was reported that the wild type contributed 41% variance by the first three eigenvectors to the total motion. For D49N, Y64S, W68G, and F94A, variance contribution by the first three eigenvectors is 55, 41, 63, and 32%, respectively. These results, particularly the D49N and W68G mutations, are significantly in uniformity with the RMSD, RMSF, and Rg results because these two mutations significantly affected the overall dynamics of the proteins and PZA binding.

**FIGURE 6:**
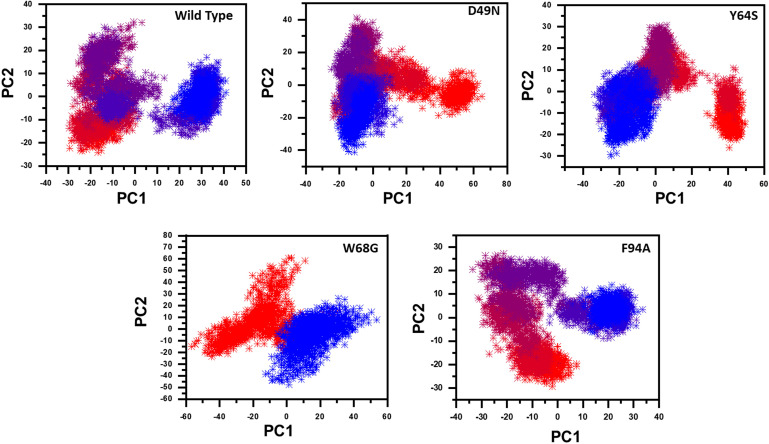
Principal component analysis of all systems, including the Wild type and the four mutants. The first two principal components (PC1 and PC2) are used to project motion in the space phase at 300 K.

We further plotted the principal components (PC1 and PC2) to cluster the trajectories motion for a perusable understanding. The conformational transition from one to another is represented in different colors (red to blue). Given in Figure 6, each dot represents a single frame from the trajectory. The mutant complexes variable phase space as compared to the WT. Together, all these results indicate that mutations significantly affect the structure that has led to the resistance against PZA drug.

### Destabilization of Fe^2+^ Ion by Mutations Induced Conformational Changes

**FIGURE 7:**
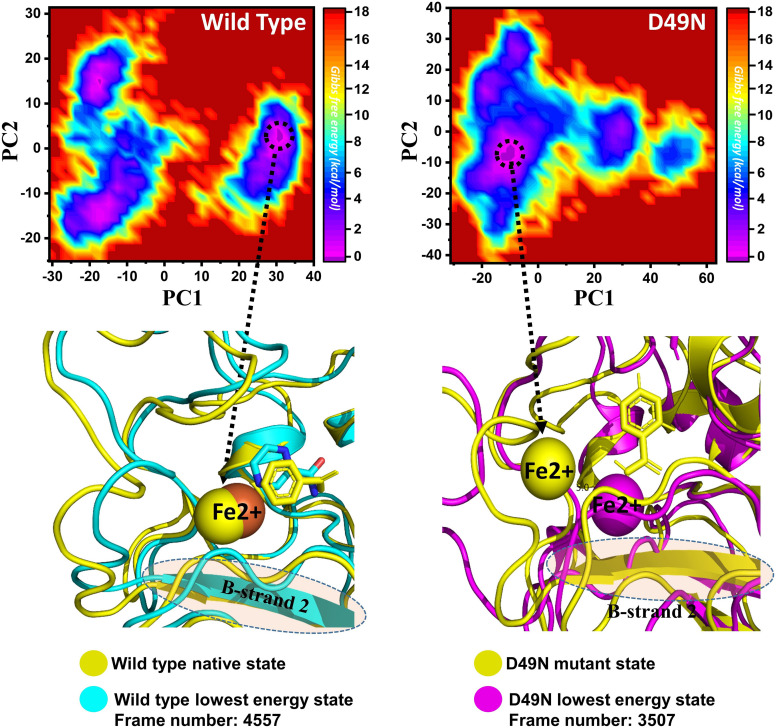
Structural rearrangement of Fe^2+^ and the other regions in the protein given above (WT and D49N mutant). The lowest energy conformation from the wild type (92 ns) for the D49N (70 ns) was extracted and compared with the native state. The circle represents the lowest energy conformation.

Three histidine residues and one asparagine residue coordinate the Fe^2+^ ion. Li/Merz ion parameters for divalent Fe^2+^ ion was used to generate the topology. Mutations induced by Fe^2+^ destabilization during the simulation were determined by using the free energy landscape. It was found that Fe^2+^ is greatly influenced by the mutations. As given in RMSD and RMSF that the stability of each system is differentially affected, while the residual flexibility also showed variations. As presented in Figure 7, in the wild type structure, the Fe^2+^ did not move out significantly, but other regions showed little dynamic differences. The lowest energy conformation was attained at 92 ns. The only metastable state was extracted for wild type PZase is given below, which shows that the protein conformation is not altered during simulation.

On the other hand, as presented in Figure 7, the mutant system D49N showed destabilization of the Fe^2+^ ion. The structural coordinates extracted from the simulation trajectory at 70 ns represent the lowest conformation. In the case of D49N, the β-sheet two is significantly affected by transforming conformation.

**FIGURE 8:**
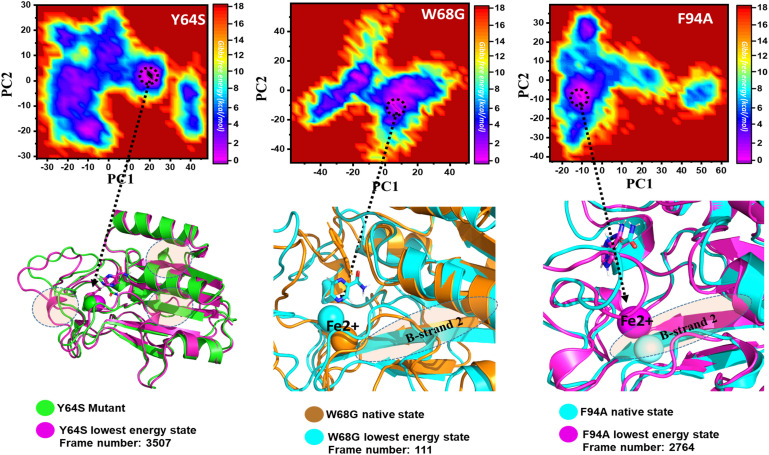
Structural rearrangement of Fe^2+^ and the other regions in the protein given above (Y64S, W68G, and F94A mutants). The lowest energy conformation from each trajectory was extracted and compared with the native state. The circle represents the lowest energy conformation.

Y64S has no significant effect on the enzyme and has the lowest energy conformation state attained at 72 ns. As given in RMSD and RMSF, the structural dynamics are not significantly affected by the Y64S mutation. All the analysis performed for Y64S in the manuscript discover consistent results and found Y64S as a comparatively less-lethal mutation than others. On the other hand, as reported above, W68G was significantly involved in structural destabilization and Fe^2+^ rearrangements. Along with the Fe^2+^ replacement and distortion of the coordination, the β-sheet 2 also flipped and thus causes a displacement of Asp49 residues that forms Fe^2+^ coordination along with the three histidine residues. The lowest conformational state of the W68G was extracted (5ns) after attaining the equilibrium (Figure 8).

### Mutation Diminishes the Binding Affinity of PZA

The MM-GBSA approach was employed to assess the binding affinity of WT and mutated receptors and ligand [1,2]. The last 10 ns trajectory, 500 snapshots, were used as input to estimate dominant forces between the protein and ligand interactions. The total binding free energies ΔGbind of WT and mutants (WT/-8.13, D49N/-5.93, Y64S/-4.88, W68G/-4.02, and F94A/-4.03) were calculated in kcal/mol (Table 2). The total energies of mutants compared to the WT indicates that these mutations drop the binding strength of the PZA. The vdW, Elec, and ΔPS energies contribution to the binding energies of the mutants compared to the WT were significantly low. It explores that the mutated proteins have weak binding to PZA. Mutations that are not involved in the direct interaction with the PZA affect orientation coordination of active site residues involved in direct contact with the PZA.

**TABLE 2:** shows the binding affinity comparison between the wild type and mutant systems.

Complex	vdW	Elec	Δ_*PS*_	SASA	MMGBSA	Δ_*TS*_	ΔG_*bind*_
Wild	−19.25	−23.37	23.48	−3.19	−20.25	−12.12	−8.13
D49N	−16.46	−17.30	19.15	−6.54	−17.21	−11.28	−5.93
Y64S	−18.23	−19.58	17.11	−9.27	−18.05	−13.17	−4.88
W68G	−15.88	−17.21	13.54	−11.10	−15.25	−11.23	−4.02
F94A	−17.14	−15.23	8.22	−10.01	−13.32	−9.73	−4.03

*All the energies are given in kcal/mol.*

## Discussion

Different studies have revealed that the administration of PZA, along with RIF and INH, is efficacious in treating Mtb infections (Gu et al., 2016; Khan et al., 2018c). Mtb resistance to these drugs renders front-line therapy ineffective, and as a consequence TB patient are exposed to a higher dose of the drugs. This leads to strong side effects on the patients and lower survival chances (Akhmetova et al., 2015; Khan et al., 2018d). Mitchison (1985), Miotto et al. (2014, 2017), Shi et al. (2014), Junaid et al. (2018), and Khan et al. (2020f). Since 1972, PZA was used as an active drug against the Mtb by targeting *panD* gene and had played key role in clearing persistent Mtb. Mutations in pncA, resulting in a loss of function of PZase, represent the primary molecular mechanism for PZA resistance in clinical strains. Pyrazinoic acid (POA) binds to the *pncA* active site and any conformational changes efflux the drug from the active site and this compromise the activity of the drug (Yadon et al., 2017). Additionally, the POA binding pocket is relatively small so any conformational changes result in unviability of the drug (Hewlett et al., 1995). It is clear that how the conformational changes affect the PZA binding, so, in this study, we selected D49N, Y64S, W68G, and F94A mutations at the Fe^2+^ binding site and PZA binding of PZase for their possible role in resistance to PZA (Miotto et al., 2017). We determined how the conformational changes may affect the binding of PZA and hinder the treatment of Mtb and eventually lengthen the eradication of tuberculosis.

In this regard, *in silico* techniques such as MD simulations were utilized to study the said mutations role in PZase resistance to PZA. These methods are widely used to understand the mechanism of resistance and any binding perturbation caused by mutations (Khan et al., 2020c,e, 2021a,b). It unveils conformational changes of proteins caused by any intrinsic mutations or ligand binding. This information is vital for devising novel strategies to combat drug resistance strains (Khan et al., 2019c, 2020b). Initial investigation of our selected mutations revealed that these mutations had altered the binding affinity of the PZA drug which shows that these mutations have clear role in resistance. Further characterization using biophysical tools revealed that RMSD and RMSF values suggest smaller fluctuations in wild type and higher fluctuations in mutant types. This probably suggest that wild type is highly stable, whereas more fluctuations in mutant type during the course of simulation suggest that the selected mutations are classified as highly destabilizing, and these findings are in line with previous experimental studies conducted on native and mutant (Q10P, D12A, G97D, R123P, T76P, G150A, H71R, W68R, W68G, and K96R). They reported that the mutations causes structural flexibility and thus weaken the drug binding (Khan et al., 2019b). The RMSF high fluctuation in mutant as compared to the WT discloses that the mutations have profound effect on the binding of the drug to the active site of the protein. A previous study carried out by *Muhammad et al*. also concluded that mutations in the PZA enzyme affect the binding orientation of PZA drug by shortening active pocket volume (Junaid et al., 2018, 2020; Khan et al., 2019c, 2020d). Findings of the current study may also suggest that said mutations affect the binding pocket, due to which the binding pocket volume as a whole is disturbed. Any distortion in the functional cavity volume might alter the binding affinity of PZA. This supports the previous study carried out by Vats et al. that mutations at the active pocket decrease the optimum affinity of the drug (Junaid et al., 2018, 2020). The residual flexibility also showed that each mutation displays a different frequency of fluctuations. Conformational dynamics, such as principal component analysis and free energy landscape, which are handy techniques reported by other studies, explored that the binding of Fe^2+^ is significantly affected. The main four residues coordinating the metal ion are disturbed during the simulation. In current study we observed different Rg pattern for the wild type and mutants. In case of wild type, initially Rg value increased and then it remains flat, whereas various patterns of increase/decrease were observed for all the mutants. These patterns suggest that the internal dynamics of each system is impacted by the mutation and eventually contributed to the PZA resistance. This notion is also supported by previous study (Jamal et al., 2020; Karmakar et al., 2020). The lowest energy minima conformation from each trajectory was extracted and compared with the native state that reported significant variations in Fe^2+^ binding and β–stands 2 specifically also confirmed by published literature (Khan et al., 2019c; Junaid et al., 2020). Analogous results have been reported that demonstrate that Fe^2+^ position is affected by catalytic and non-catalytic residues mutation. Furthermore, The Gibbs free energy to estimate the impact of the said substitutions on the binding of PZA. It was witnessed that mutations have significantly reduced the binding affinity of PZA and D49N and W68G being the major which contribute significantly to PZA resistance (Rehman et al., 2019). The study of dynamic behavior provided highly adequate knowledge on the PZase mutation that affected its structure, as well as perspectives into how conformational differences influence protein-ligand interactions which would aid the development of structure-based drug designing against the PZA target of Mtb.

In conclusion, we performed extensive MD simulations in triplets to explore the impact of D49N, Y64S, W68G, and F94A mutations on the PZase resistance to PZA. Our analysis revealed that these mutations affect stability, internal structural dynamics, and the binding energy of PZA. Our study further suggests that the stabilization of Fe^2+^ and β–stand 2 were affected. Hence, there is a dire need to design more potent drugs that would potently inhibit Mtb.

## Data Availability Statement

The original contributions presented in the study are included in the article/Supplementary Material, further inquiries can be directed to the corresponding author/s.

## Author Contributions

AN, AK, SU, SSA, and SA conceptualized the study. AK, SS, SY, SHA, and MA did the analysis. AK, MA, and SSA draft the manuscript. AN, AK, SHA, and D-QW revised and finalized the manuscript. LA performed the revision and writing improvement. D-QW is an academic supervisor and supervised the study.

## Conflict of Interest

The authors declare that the research was conducted in the absence of any commercial or financial relationships that could be construed as a potential conflict of interest.

## Footnotes

1 http://biosig.unimelb.edu.au/mcsm/
