# Supplementary material for: Insights Into Mutations Induced Conformational Changes and Rearrangement of Fe2+ Ion in pncA Gene of Mycobacterium tuberculosis to Decipher the Mechanism of Resistance to Pyrazinamide
Source: Front Mol Biosci. 2021 May 20;8:633365. doi: 10.3389/fmolb.2021.633365 (PMC8174790; doi:10.3389/fmolb.2021.633365)
Supplement: Supplementary file 1 [file Data_Sheet_1.docx]

**Supplementary Materials**

**Insights into mutations induced conformational changes and rearrangement of Fe^2+^ ion in *pncA* gene of *Mycobacterium Tuberculosis* to decipher the mechanism of resistance to pyrazinamide**

Asma Sindhoo Nangraj^1^, Abbas Khan^1^, Shaheena Umbreen^2^, Sana Sahar^3^, Maryam Arshad^4^, Saba Younas^5^, Sajjad Ahmad^6^, Shahid Ali^7^, Syed Shujait Ali^7^, Liaqat Ali^10^, Dong-Qing Wei^1,8,9*^

^1^Department of Bioinformatics and Biological Statistics, School of Life Sciences and Biotechnology, Shanghai Jiao Tong University, Shanghai, 200240, P.R. China.

^2^Department of Botany, University of Okara, Okara, Punjab, Pakistan.

^3^ The Islamia University of Bahawalpur, Pakistan.

^4^Government College University Faisalabad, Sahiwal, Pakistan.

^5^University of Education, Lahore, Pakistan.

^6^National Center for Bioinformatics, Quaid-i-Azam University, Islamabad, Pakistan.

^7^Center for Biotechnology and Microbiology, University of Swat, Swat, KP, Pakistan.

^8^State Key Laboratory of Microbial Metabolism, Shanghai-Islamabad-Belgrade Joint Innovation Center on Antibacterial Resistances, Joint International Research Laboratory of Metabolic & Developmental Sciences and School of Life Sciences and Biotechnology, Shanghai Jiao Tong University, Shanghai 200030, P.R. China

^9^Peng Cheng Laboratory, Vanke Cloud City Phase I Building 8, Xili Street, Nashan District, Shenzhen, Guangdong, 518055, P.R China.

^10^Department of Biological Sciences, National University of Medical Sciences, Islamabad, Pakistan.

***Corresponding Author Information**

**Professor Dr. Dong-Qing Wei**

[**dqwei@sjtu.edu.cn**](mailto:dqwei@sjtu.edu.cn)

**
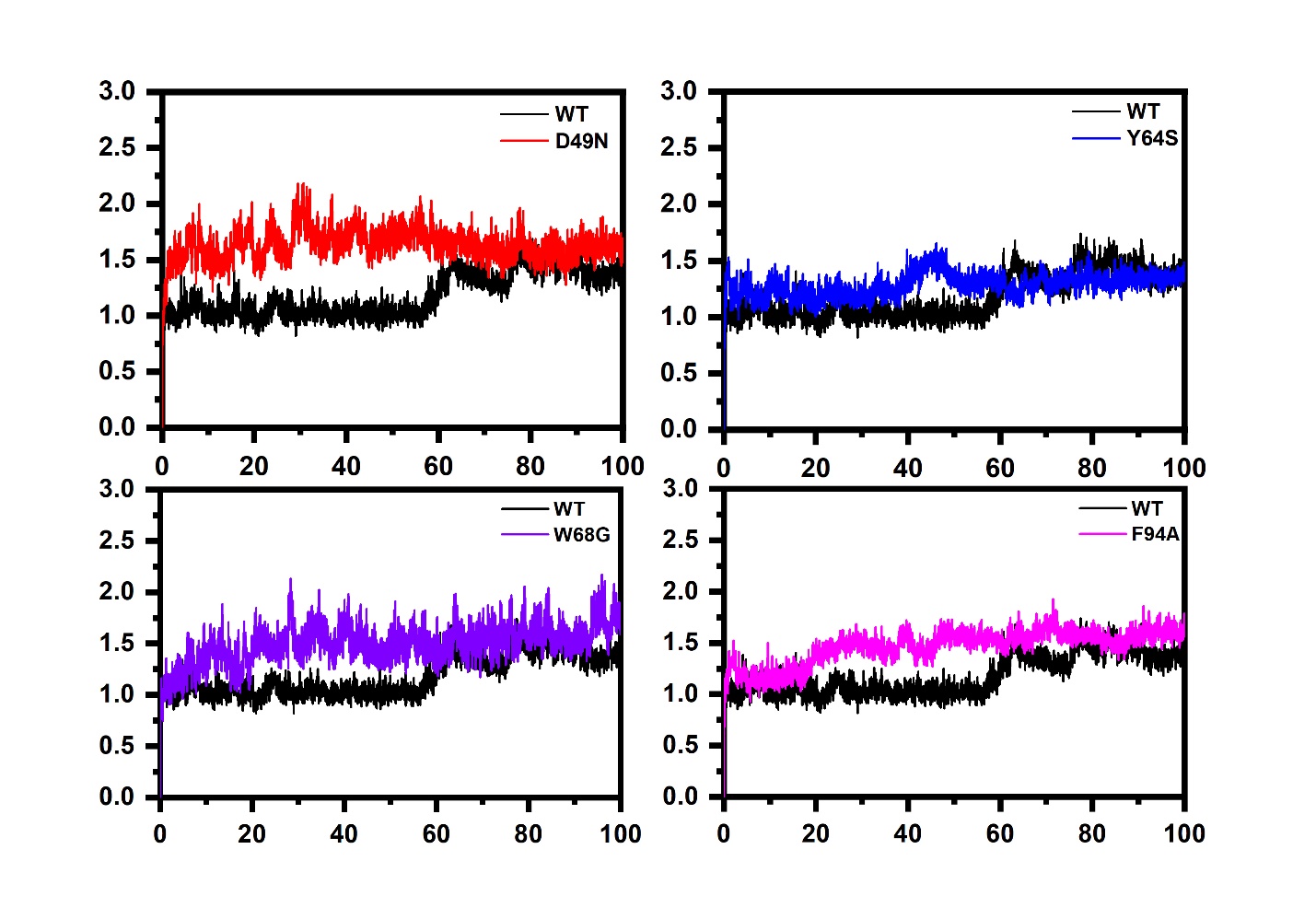
**

**Figure S1:** RMSD of the replicate 2.

**Figure S2:** RMSD of the replicate 3.

**
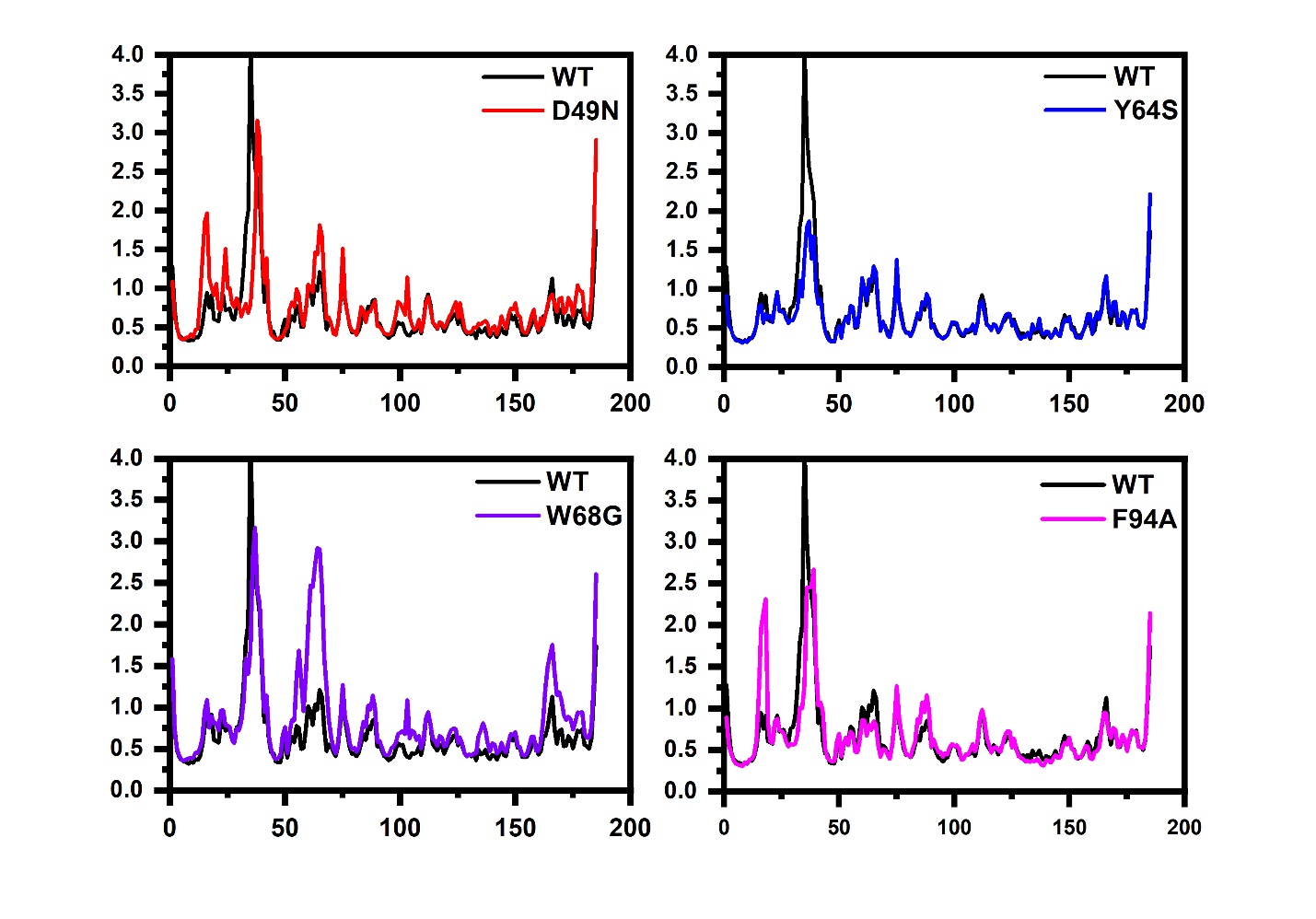
**

**
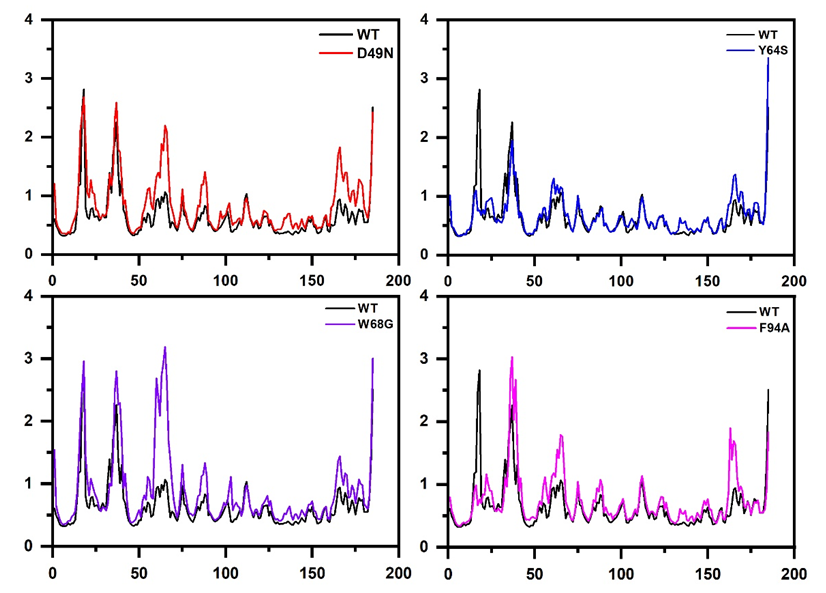
Figure S3:** RMSF of the replicate 2.

**
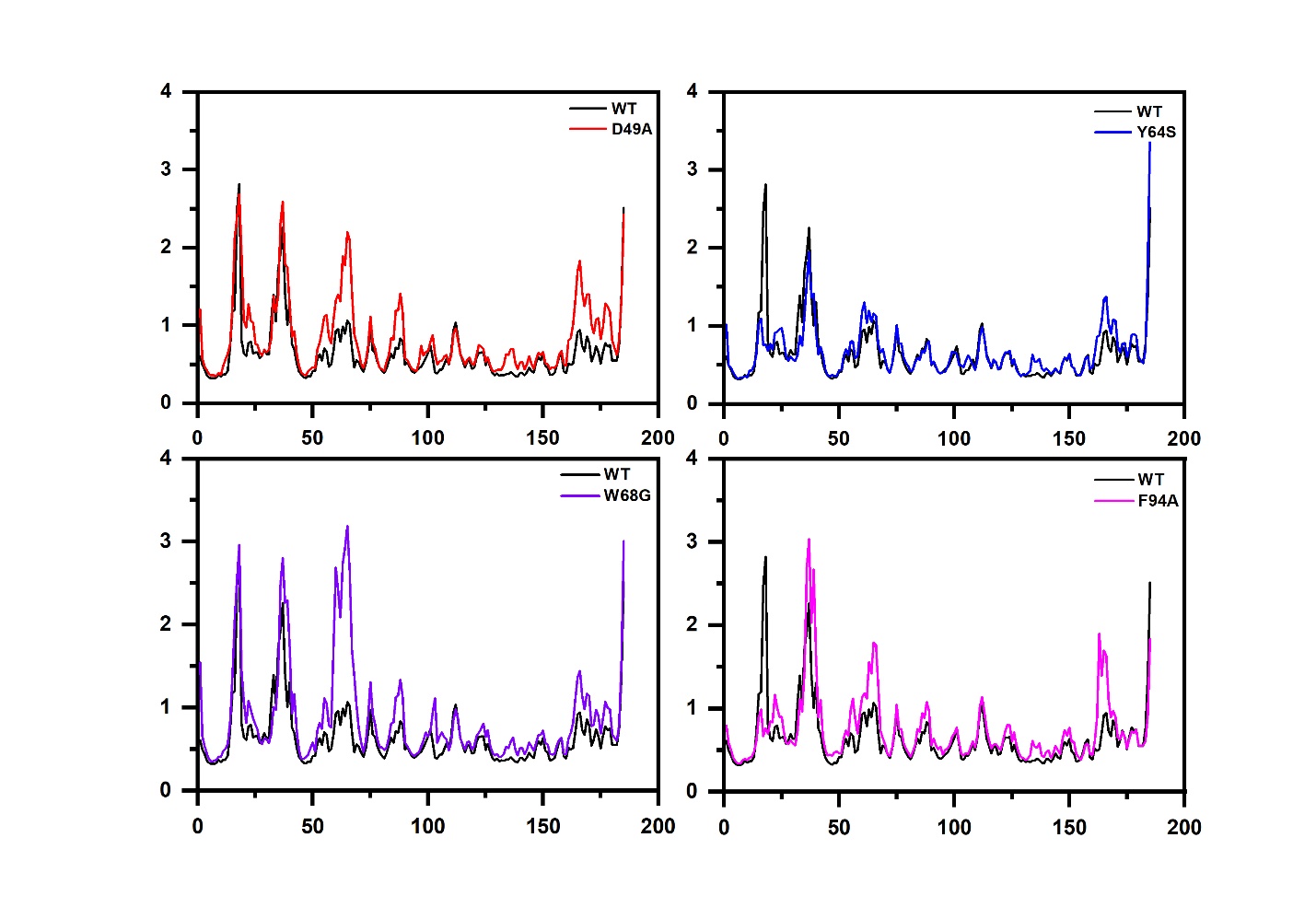


Figure S4:** RMSF of the replicate 3. ****

**Figure S5:** showing the distance between the receptor and PZA molecule. The distance between the Oxygen atom of the PZA at tail and Cys138 residue which is important active site residue.

**Figure S6:** Rg of the replicate 1. The x-axis show time in nanoseconds while y-axis represent Rg in Å.

**Figure S7:** Rg of the replicate 2. The x-axis show time in nanoseconds while y-axis represent Rg in Å.

**Figure S8:** Rg of the replicate 3. The x-axis show time in nanoseconds while y-axis represent Rg in Å.
